# Adult entero‐enteric intussusception secondary to lipoma

**DOI:** 10.1002/ccr3.2460

**Published:** 2019-09-27

**Authors:** Mohammad Hanafiah, Mardiana Abdul Aziz, Siti Mayuha Rusli

**Affiliations:** ^1^ Department of Radiology Faculty of Medicine Universiti Teknologi MARA Sungai Buloh Selangor Malaysia; ^2^ Institute for Pathology, Laboratory and Forensic Medicine Sungai Buloh Selangor Malaysia; ^3^ Department of Pathology Faculty of Medicine Universiti Teknologi MARA Sungai Buloh Selangor Malaysia; ^4^ Department of General Surgery Faculty of Medicine Universiti Teknologi MARA Sungai Buloh Selangor Malaysia

**Keywords:** computed tomography, intussusception, lipoma, small bowel, ultrasound

## Abstract

Diagnostic imaging methods are normally required to make the preoperative diagnosis of adult intussusception. Furthermore, it helps to define the location and nature of the associated mass as lead point if present. Lipoma may appear as lesion of fat attenuation expressed in Hounsfield unit on CT scan.

## Graphical abstract

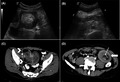

## 1 CASE

**Figure 1:**
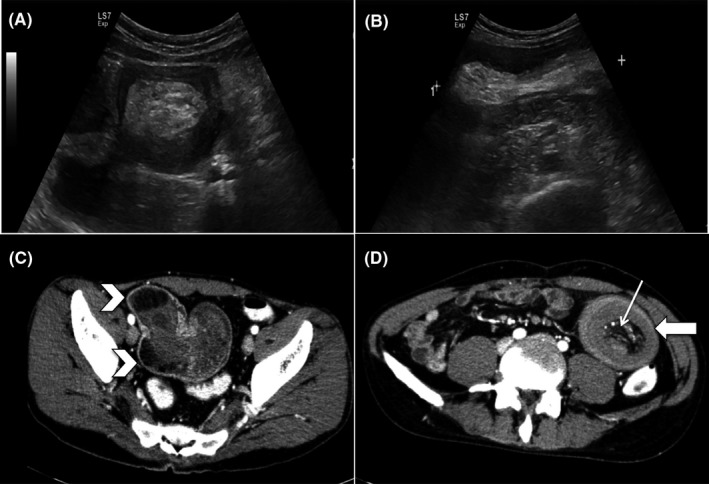
Selected ultrasound (A & B) and CT (C & D) images demonstrating an entero‐enteric intussusception. The ultrasound images reveal characteristic features of “target” sign on transverse view (A) and “pseudokidney” sign (B) on longitudinal view. The CT scan demonstrates “target”‐shaped soft tissue mass with layering effect (white thick arrow). The characteristic intraluminal mesenteric vessels (thin white arrow) are evident. Note is made of a few well‐defined lesions of fat density (−50 to −30 HU) representing lipomas at the most proximal part of the intussusceptum acting as the lead point

**Figure 2:**
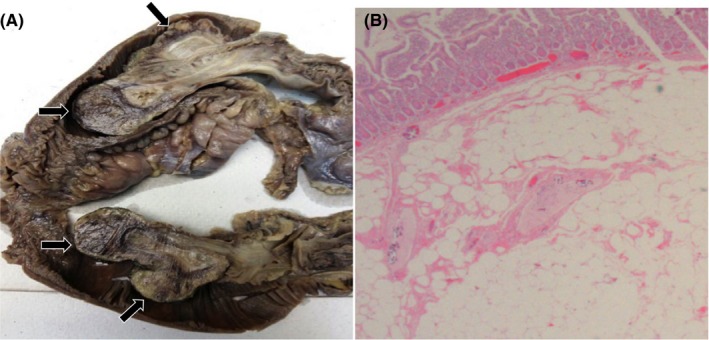
The gross specimen (A) of the small bowel resection, which is split in half, demonstrated an entero‐enteric intussusception. The lipomatous polyps are shown (black arrows). The histological analysis (B) revealed collection of mature adipocytes in the submucosa consistent with lipomas (H&E, 4× magnification)

A 35‐year‐old male patient presented with intermittent colicky abdominal pain and postprandial vomiting. He was dehydrated, and his abdomen was soft and nondistended. He had low‐grade pyrexia (37.8°C) and elevated total white cell count (25.1 × 10^9^/L) and CRP (14.8 mg/dL). An abdominal ultrasound showed dilated fluid‐filled small bowel loops associated with “target” signs in the left lower abdominal region indicating the presence of an intussusception (Figure 1). An abdominal contrast‐enhanced CT revealed an entero‐enteric intussusception with a few lesions of fat density (−50 to −30 HU) at the most proximal part of the intussusception (Figure 1). Laparotomy and small bowel resection confirmed intussusception with lipomatous polyps (1.5‐2.0 cm) acting as the lead point (Figure 2).

Unlike in children, adult intussusception is usually caused by a tumor acting as the lead point. Lipoma is one of the common causes.^1^ Preoperative diagnosis of adult intussusception is very challenging due to nonspecific nature of the symptoms. Imaging is usually required to arrive at the diagnosis. On ultrasound, the classical features include “target” or “doughnut” signs on transverse view and “pseudokidney” sign on longitudinal view.^2^ Abdominal CT is the most sensitive radiological method to confirm intussusception. The characteristic features include a “target” soft tissue mass with a layering effect associated with the presence of mesenteric vessels within the bowel lumen.^2^ If the underlying mass is identified, CT scan can define the location, the nature of the mass, and its relationship with the surrounding tissues.^2^

## CONFLICT OF INTEREST

None declared.

## AUTHOR CONTRIBUTIONS

MH: involved in manuscript preparation and drafting and editing of the manuscript. MAA and SMR: involved in drafting and editing of the manuscript. All the authors approve the submitted manuscript.
